# On the Core Prescriptions and Their Mechanisms of Traditional Chinese Medicine in Hepatitis B, Liver Cirrhosis, and Liver Cancer Treatment

**DOI:** 10.1155/2022/5300523

**Published:** 2022-09-23

**Authors:** Zhendong Wang, Yong Zhang, Qiuyun Zhang, Qiang Ao, Changyong Luo, Bochuan Wang, Chen Bai, Xueyi Ge, Yuhan Wang, Jing Wang, Ying Qian, He Yu, Xiaohong Gu

**Affiliations:** ^1^School of Traditional Chinese Medicine, Beijing University of Chinese Medicine, Beijing, China; ^2^Liver Disease Branch, Shandong Hospital of Traditional Chinese Medicine, Jinan, China; ^3^Capital Medical University, Beijing, China; ^4^Beijing Tcmages Pharmaceutical Co., Ltd., Beijing, China; ^5^Dongfang Hospital, Beijing University of Chinese Medicine, Beijing, China; ^6^Shandong University of Traditional Chinese Medicine, Jinan, China

## Abstract

**Background:**

As a frequent cause of death in cancer patients, liver cancer usually occurs in hepatitis B and cirrhosis. In China, Chinese people have been using traditional Chinese medicine (TCM) in treating various chronic liver diseases, which could effectively improve the symptoms and slow down the progression of liver diseases. However, due to the complexity rules of TCM prescription, their action mechanisms are still not clearly understood, which may affect the popularization of effective prescriptions. This study aims to identify the core TCM herbs in the treatment of hepatitis B, liver cirrhosis, and liver cancer so as to clarify the mechanism of action of the core herb networks.

**Methods:**

There were 1,673 prescriptions for chronic liver diseases collected in this study, of which 854 were hepatic B prescriptions, 530 were for liver cirrhosis, and 289 were for liver cancer. The basic characteristics of herbal medicine were firstly explained via descriptive analysis, then the core prescriptions of herbal medicine were analyzed through association rule, and finally, the mechanism of core prescriptions was explored with the help of systematic network pharmacology and by applying such databases as TCMIP, HERB, OMIM, GeneCards, KEGG, and software like RStudio and Cytoscape.

**Results:**

The rule of the core prescriptions in these cases was characterized by the application of herbs with both cold and warm properties, in which bitter herbs with cold property took priority. Tonifying deficiency, clearing heat, and activating blood circulations to remove stasis were common treatment principles for the three liver diseases. Turmeric Root Tuber (YuJin), White Peony Root (BaiShao), Bupleurum (ChaiHu), Salvia miltiorrhiza (DanShen), and Astragali Radix (HuangQi) were prescribed the most in hepatitis B treatment to invigorate the spleen and soothe the liver. Astragali Radix (HuangQi), Tuckahoe (FuLing), Atractylodis Macrocephalae Rhizoma (BaiZhu), Fructus Polygoni Orientalis (ShuiHongHuaZi), and Curcumae Rhizome (EZhu) were most frequently applied in liver cirrhosis treatment to replenish qi and activate blood. *Oldenlandia* (BaiHuaSheSheCao), Bearded Scutellaria (BanZhiLian), Curcumae Rhizome (EZhu), and Cardamom (DouKou) were most frequently prescribed to eliminate cancer toxin, invigorate the spleen, and activate blood. These core herbs mainly act through signal transduction and immune system pathways, in which the PI3K-Akt pathway plays a key role. The core prescription for liver cirrhosis regulated more endocrine system pathways than the hepatitis B prescription, and liver cancer prescription regulated more nervous system-related pathways.

**Conclusion:**

Three core prescriptions for hepatitis B, liver cirrhosis, and liver cancer treatment were identified, which acted mainly through signal transduction and immune system pathways to regulate immunity and cell growth and participate in inflammation inhibition, in which liver cancer prescription regulated more pathways, especially more nervous system-related pathways than the other two.

## 1. Introduction

The global morbidity and mortality rate of liver cancer has been increasing in the past years, which has become the third most common cause of death from tumors [1]. Asia and Africa have the highest incidence rate of it in the world [2]. It was reported by Surveillance, Epidemiology, and End Results (SEER) that liver cancer has become the fastest growing cause of cancer-related death in the United States ever since the beginning of the 21st century [3]. It was estimated that more than 1 million people worldwide would be affected by liver cancer each year by 2025. Hepatitis B virus infection is the main risk factor for the progress of liver cancer, accounting for 60% of primary hepatocellular carcinoma cases in Asia and Africa [4–6]. The long-term liver inflammation caused by hepatitis B virus infection triggers compensatory liver repair and regeneration and ultimately leads to liver fibrosis or cirrhosis [7]. This pathological state plays an important role in the precancerous environment of the liver; more than 80% of hepatocellular carcinomas occur in the fibrotic or cirrhotic liver [8]. It is known that the incidence and progression of hepatitis B, cirrhosis, and liver cancer are correlated to a certain degree, and the medical and family burden they brought about is gradually increasing. Therefore, it is of great significance to block the progression of liver diseases and reduce the social burdens they incur.

With the development of complementary and alternative medicine around the world, the role of traditional Chinese medicine (TCM) in tumor prevention and treatment has drawn more and more attention. Over 60% of the antitumor medicines were developed from natural herbal products these years [9]. In China, TCM has been widely used in liver cancer treatment combined with chemotherapy, radiotherapy, surgical resection, and liver transplantation and even used alone in the middle and late stages of liver cancer [10, 11]. TCM participation could reduce the adverse reactions of conventional treatment, relieve symptoms, protect liver function, and improve overall survival [12–14]. Studies have shown that, in hepatitis B cases, patients who received TCM treatment had a significantly lower risk of liver cirrhosis than those who did not [15]. TCM intervention after hepatitis B and liver cancer surgery was clinically proved to be able to prevent the occurrence of liver cancer [16, 17]. Compared with applying antiviral herbs alone in the treatment of liver fibrosis, a combining application of TCM herbs could increase the reversal rate of liver fibrosis, reduce the risk of liver cancer [18], and effectively alleviate the symptoms caused by liver dysfunction [19].

By analyzing the application characteristics, the prescription rules of the herbs, and constructing diagrams, data mining could help to discover the potential correlation between herbs, which might provide support for doctors in developing treatment protocols [20]. Analysis of frequency statistics and association rules is a common technical method of it [21, 22]. Studies have identified the core prescriptions through retrospective analysis of patients with chronic kidney diseases, as well as prescription frequency and association rules analysis, and have explored the TCM pathogenesis of chronic kidney diseases [23].

The “multicomponent and multitarget network” in systematic pharmacology happened to coincide with the holism concept of TCM prescription, which might effectively bridge the gap between modern medicine and TCM and provide new research methods for TCM theories and would greatly promote the synergistic development between TCM and modern medicine [24]. By analyzing the compound targets and the enrichment pathways of TCM prescriptions, the mechanism of core prescriptions would be studied further. Some researchers have applied data mining combined with network pharmacology in exploring new prescriptions for TCM treatment of recurrent respiratory infections and demonstrated the mechanism of action of the core prescriptions [20].

**Figure 1:**
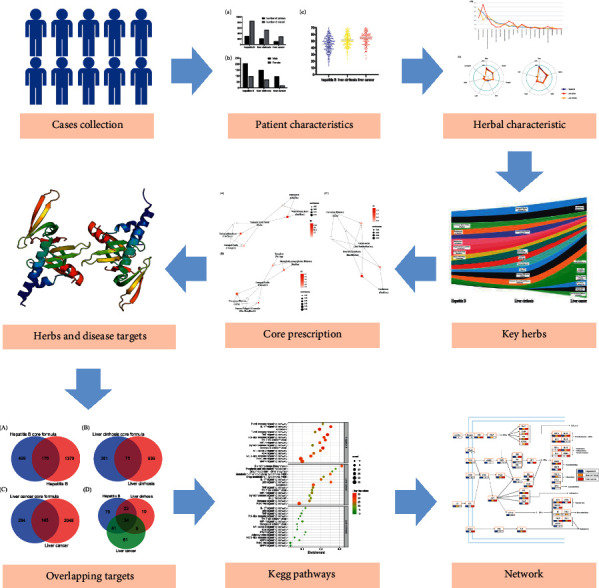
Workflow.

This study proposed a comprehensive method based on data mining and systematic pharmacology, analyzed the rules of TCM prescriptions, and revealed their mechanisms. Clinical data of patients with liver diseases from the two centers were collected, and the prescriptions of these cases were analyzed by descriptive statistics and association rules to explore the prescription rules and core prescriptions in the cases. The important targets were retrieved from the database, and Metascape and KEGG were used to further analyze the biomarker pathways. Workflow is shown in Figure 1. The findings of this study would help us to identify the core herbs and prescriptions and analyze the mechanism of action of the core prescriptions, thus providing a deeper understanding of TCM prescriptions and promoting the development of medicine for chronic liver disease treatment as well as TCM studies in the future.

## 2. Materials and Methods

### 2.1. Data Collection and Processing

In this study, 1,673 prescriptions from the outpatient departments of the Affiliated Hospital of Shandong University of Traditional Chinese Medicine by Professor Changjian Yin and the Affiliated Hospital of Capital Medical University by Professor Ying Qian were analyzed retrospectively from January 2020 to June 2021, among which, 854 cases were diagnosed as chronic hepatitis B, 530 were cirrhosis, and 289 were liver cancer.

Hepatitis B diagnostic criteria referred to the guidelines for the prevention and treatment of chronic hepatitis B [25]. Diagnostic criteria included HBsAg and HBV-DNA (+) in serum samples. Inclusion criteria included those who met the diagnostic criteria. Exclusion criteria included ① simultaneous infection with HIV, HCV, and HDV; ② those who suffered from liver cirrhosis, liver cancer, and other liver diseases; ③ patients with severe mental and other internal organ diseases.

The diagnostic criteria of decompensated liver cirrhosis referred to the Chinese guidelines on the management of liver cirrhosis [26]. Diagnostic criteria were as follows: ① Histology results should meet the diagnosis of liver cirrhosis. ② There should be complications related to portal hypertension, such as ascites and gastric varices. Inclusion criteria included patients who met the diagnostic criteria. Exclusion criteria included ① liver cancer; ② serious cardiovascular, renal, or mental diseases or coagulation dysfunction.

The diagnostic criteria of primary liver cancer referred to the diagnostic criteria of the guidelines for diagnosis and treatment of primary liver cancer [27]. Diagnostic criteria were as follows: it should be confirmed by imaging examination, pathological examination, and clinical manifestations. Inclusion criteria included patients who met the diagnostic criteria. Exclusion criteria were as follows: ① patients with metastatic tumor; ② patients with a history of surgery; ③ patients with serious cardiovascular, renal, or mental diseases, with coagulation dysfunction, and so on.

The general clinical information and the information on Chinese herbal medicine prescriptions of these cases were collected, including the patients' age and gender and the formulae prescribed to them. The data were entered into Microsoft Office Excel, and the database was subsequently established. The herbs in the database were further standardized, and their properties, flavors, and efficacies of the herbs were completed according to the National Pharmacopoeia Commission and Chinese Materia Medica. Two researchers completed the data entry separately.

This study was reviewed by the Ethics Committee of Beijing University of Chinese Medicine, with ethical batch number 2019BZHYLL0204, which was strictly implemented in accordance with the ethical system.

### 2.2. Analysis of Basic Information and the Characteristics of Prescriptions

Microsoft Office Excel software was used to statistically analyze the distribution of patients' visits, age, and gender, and GraphPad Prism was used for visual display. Firstly, a general descriptive analysis was performed on the properties, flavors, and efficacies of the herbs in the prescriptions. Then the properties, flavors, and other aspects of the high-frequency herbs were analyzed and visually displayed so as to comprehensively show the therapeutic ideas of the prescriptions. Finally, association rules were used to screen the core herbs with strong association.

#### 2.2.1. Descriptive Analysis of the Properties, Flavors, and Frequency of the Herbs

Descriptive analysis was performed on the characteristics (the properties, flavors, and categories) of the herbs. The siqi (four properties) included five items: cold, heat, warm, cool and mild, and the wuwei (five flavors) included seven items: sour, bitter, sweet, pungent, salty, astringent, and light. The herbs were divided into 21 categories according to their efficacies, which are deficiency-tonifying herbs, heat-clearing herbs, blood-activating and stasis-removing herbs, damp-draining herbs, qi-regulating herbs, exterior-relieving herbs, aromatic damp-resolving herbs, phlegm-resolving, cough-suppressing and panting-calming herbs, digestant herbs, blood-stanching herbs, wind-damp-dispelling herbs, tranquilizing herbs, liver-wind-calming herbs, astringent herbs, interior-warming herbs, purgative herbs, detoxification, parasiticide and itching-relieving herbs, worm-expelling herbs, resuscitative herbs, emetic herbs, and suppuration and granulation-promoting herbs. The top 10 core herbs in terms of frequency used in the three diseases were chosen for visual analysis of their properties, flavors, and proportion of use in all cases. RStudio 3.6.1 software and Mul-chart software were applied for analysis and visual display.

#### 2.2.2. Association Rules on the Screening of Core Prescriptions

Apriori algorithm was a frequent itemset algorithm that formed the association rules, which was used to analyze the clear rules in TCM treatment and obtain the core herbs in this study. Each herb was treated as an itemset, each prescription was treated as a transaction, and they were used to find out the frequent itemset, to mine the association rules between the herbs in the prescriptions, and to discover the core prescriptions. RStudio 4.0.2 software was applied for analysis and visual display.

### 2.3. Analysis of Therapeutic Mechanisms of Prescriptions

#### 2.3.1. Acquisition of Core Herb Targets

In this study, the integrative medicine pharmacology research platform TCMIP v2.0 (https://www.TCMIP.cn/TCMIP/index.php/Home/Login/login.html) [28] and the HERB database (https://herb.ac.cn/) [29] were used to gain the molecular targets of the core prescriptions and merge the obtained targets to get the relevant herb targets.

#### 2.3.2. Acquisition of Disease-Related Targets

Relevant targets for the three diseases were collected via the integration of the GeneCards database (https://www.genecards.org/) and Online Mendelian Inheritance In Man (OMIM) database (https://www.omim.org/). A score ≥10 was set in the GeneCards database to select the targets of high correlation with the disease, and the obtained targets in the two databases were merged with each other to screen out the disease-related targets.

### 2.4. Construction of PPI Network

The overlapped targets were selected as the core herbs of the relevant ones for the treatment of hepatitis B, cirrhosis, and liver cancer. The target was then processed with String (https://string-db.org/), and the species was selected as “*Homo sapiens*” to derive the data of protein-protein interaction (PPI). The resulting PPI data were imported into Cytoscape 3.8 (https://www.Cytoscape.org/) for processing the PPI network diagram.

#### 2.4.1. Functional Annotation and Enrichment Analysis

Common targets were acquired after overlapping the obtained herb targets, and Metascape (https://metascape.org/gp/index.html#/main/step1) was applied for KEGG pathway analysis. The data were uploaded as a multiple gene list, the species were selected as “*Homo sapiens*,” and the mode was set as custom analysis, *P* value <0.01, min overlap >3. After removing the disease-related pathways, each of the top 15 important pathways was selected, and the Microbiology Information website (https://www.bioinformatics.com.cn) was applied to plot the KEGG pathway bubble map.

#### 2.4.2. Construction of Core Herb Pathway Network

Enrichment analysis was applied to get the pathways, and the pathways that were related to signal transduction or immune system were retained and summarized into a Subnetwork using the Kyoto Encyclopedia of Genes and Genomes (KEGG) online tool Mapper-Search and Color Pathway. For better presentation, the intermediate genes were hidden.

## 3. Results

### 3.1. Patient Characteristics

**Figure 2:**
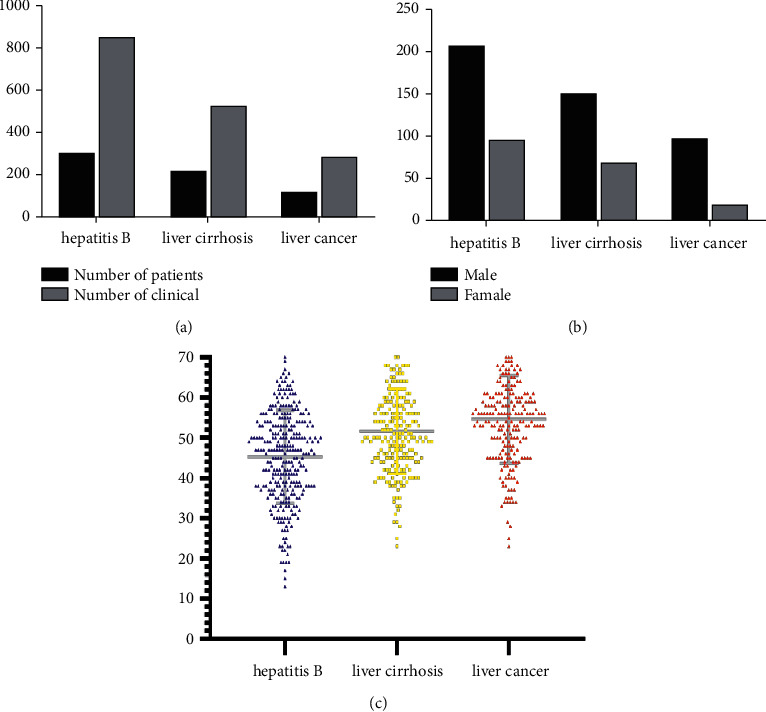
Basic information of patients. (a) Number of patients and visits. (b) Gender distribution of patients. (c) Age distribution of patients.

As shown in Figure 2, the cases with more than four herbs in a prescription were qualified and were collected in this study, involving 302 cases of hepatitis B with 205 males and 97 females, 218 cases of cirrhosis with 149 males and 69 females, and 116 cases of liver cancer with 96 males and 20 females, with an average visit of 2.8, 2.4, and 2.5, respectively. The average age of hepatitis B patients was 45.3 years, that of cirrhosis patients was 51.6 years, and that of liver cancer patients was 57.7 years. With the progression of disease, the number of patients decreased while their age increased. In terms of gender distribution, the number of male patients was higher than that of females, and the male-to-female ratios of hepatitis B, cirrhosis, and liver cancer were 2.1 : 1, 2.2 : 1, and 4.8 : 1, respectively.

### 3.2. Basic Characteristics of Herbs

**Figure 3:**
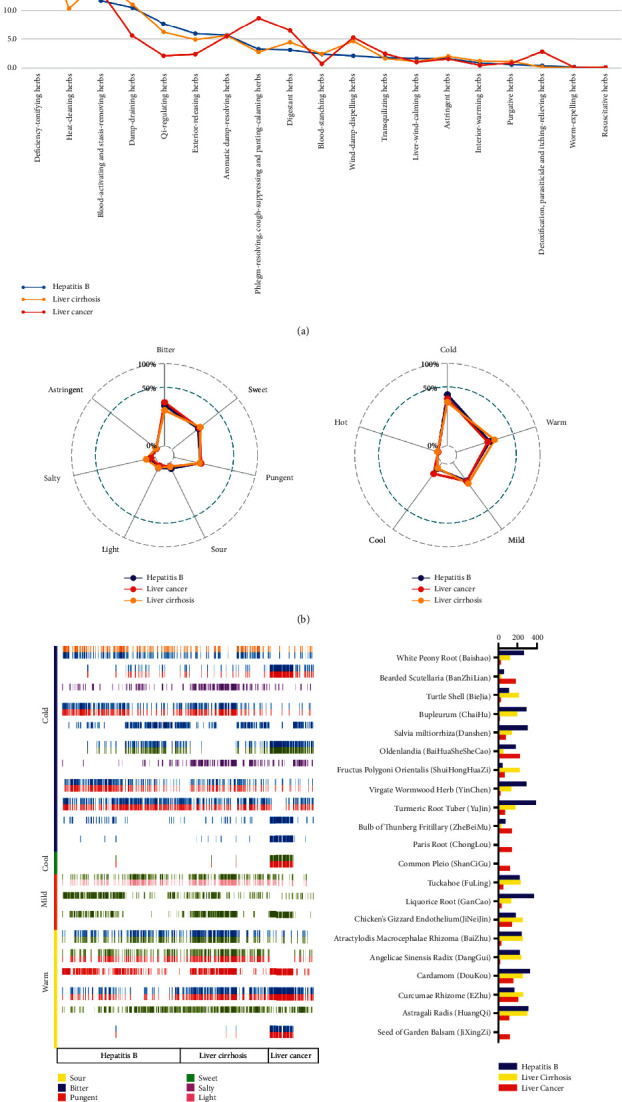
(a) The *x*-axis represents the proportion of herbs, and the *y*-axis represents the category of herb efficacies. (b) Radar chart showed the total frequency of each property in all prescriptions. (c) The *x*-axis was the name code of patients, which was divided into hepatitis B, liver cirrhosis, and liver cancer, and the *y*-axis was the herb, which was divided into cold, cool, mild, and warm according to herbal properties. The color in the figure represented the flavors of the herbs, and the histogram corresponded to herb frequency.

There were a total of 1,673 prescriptions, including 854 prescriptions for hepatitis B, 530 prescriptions for cirrhosis, and 289 prescriptions for liver cancer. The classification of herbs is shown in Figure 3(a), in which deficiency-tonifying herbs, heat-clearing herbs, and blood-activating and stasis-removing herbs were commonly used in the prescriptions for hepatitis B, liver cirrhosis, and liver cancer, accounting for more than 10%. In addition, it showed that, compared with the prescriptions for the other two diseases, qi-regulating herbs and exterior-resolving herbs were more commonly used in the prescriptions for hepatitis B. Deficiency-tonifying herbs and blood-activating and stasis-removing herbs were more commonly used in the prescriptions for liver cirrhosis, and heat-clearing herbs and phlegm-resolving herbs were more commonly used in the prescriptions for liver cancer.  Figure 3(b) showed that the cold and warm herbs were most frequently used, followed by the mild ones. As for the flavors, bitter herbs were the most used, pungent and sweet ones were of similar total frequency, and the proportion of the characteristics and the flavors of herbs used for the three diseases were basically similar.

In this study, the top 10 herbs in frequency used in three liver diseases were further extracted, with a total of 21 herbs, and their properties, flavors, and frequency of use are shown in Figure 3(c). Generally, the cold herbs were mainly bitter cold, warm herbs were mostly sweet and warm, and mild herbs were mainly sweet and mild in this study. The proportion of medication for the three diseases was basically similar. Among them, the total herb frequency for hepatitis B was 12,491 times; the highest frequency herb in it was Turmeric Root Tuber (Yujin), with a frequency of 395 times. The total herb frequency of liver cirrhosis was 8,714 times, and its highest frequency herb was Astragali Radix (HuangQi), 301 times. And for liver cancer, its total herb frequency was 4,401 times, and the highest frequency herb was *Oldenlandia* (BaiHuaSheSheCao), 227 times.

**Figure 4:**
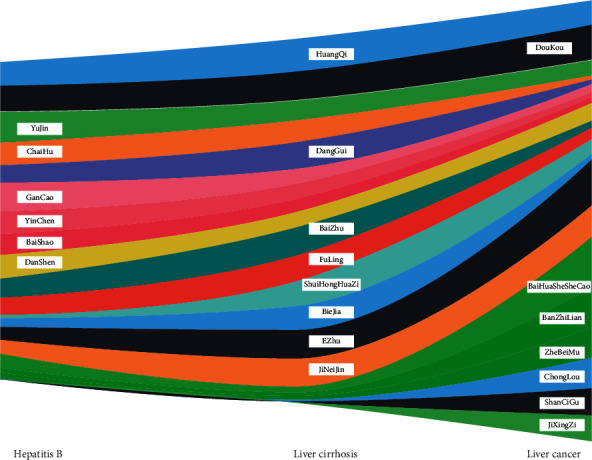
The *x*-axis is the disease, and the *y*-axis is the herb. The same color band represents a certain herb, and the width of the band represents the proportion of the frequency of the herb in a disease accounting for the total herb frequency of the disease.

Figure 4 shows the proportion of the top 10 frequently used herbs in each of the three diseases, in which Astragali Radix (HuangQi), Cardamom (DouKou), Turmeric Root Tuber (YuJin), Bupleurum (ChaiHu), Liquorice Root (GanCao), Virgate Wormwood Herb (YinChen), White Peony Root (BaiShao), Salvia miltiorrhiza (DanShen), and so on were frequently used in treating hepatitis B. Astragali Radix (HuangQi), Angelicae Sinensis Radix (DangGui), Atractylodis Macrocephalae Rhizoma (BaiZhu), Tuckahoe (FuLing), Curcumae Rhizome (EZhu), Fructus Polygoni Orientalis (ShuiHongHuaZi), Chicken Gizzard Linning (JiNeiJin), and so on were frequently used in treating liver cirrhosis. And Cardamom (DouKou), Bearded Scutellaria (BanZhiLian), *Oldenlandia* (BaiHuaSheSheCao), Curcumae Rhizome (EZhu), Paris Rhizome (ChongLou), Common Pleione Pseudobulb (ShanCiGu), and so on were frequently used in treating liver cancer.

### 3.3. Association Rules Analysis

**Figure 5:**
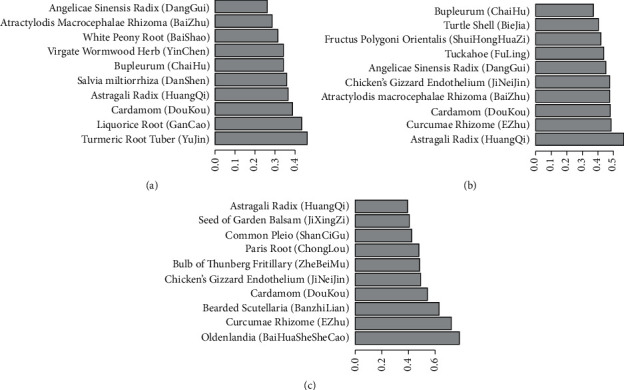
The *x*-axis is the support, and larger support represents a higher percentage and importance of the herbs. The first 10 frequency items of hepatitis B (a), cirrhosis (b), and liver cancer (c) are displayed, respectively.

**Figure 6:**
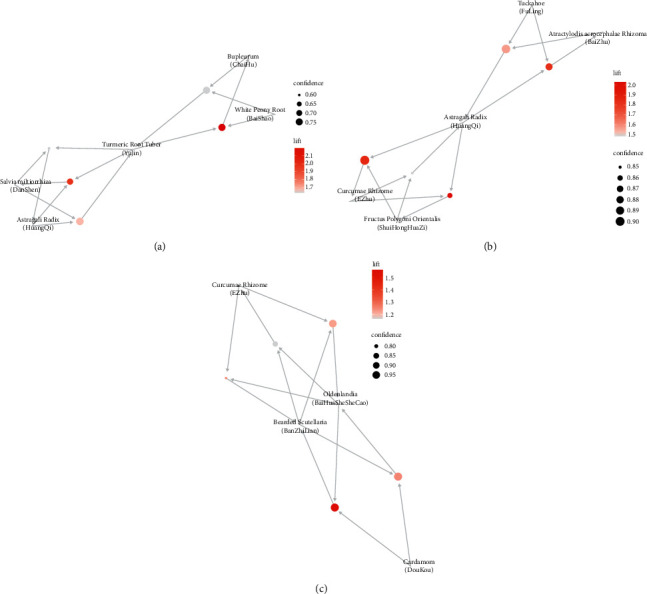
The network diagram of association rules in the first 5 support levels of hepatitis B (a), cirrhosis (b), and liver cancer (c), respectively.

Apriori algorithm was used in analyzing the association rules of the herbs in each of the three diseases. The data were firstly transformed by transactions, and the top 10 frequent items for each disease are listed in Figure 5. The top 5 rules of “support” associated with the most high-frequency herbs are shown in Figure 6 (the association rules are shown in attached file S1). It could be seen that, among the hepatitis B treatments, Turmeric Root Tuber (YuJin), White Peony Root (BaiShao), Bupleurum (ChaiHu), Salvia miltiorrhiza (DanShen), and Astragali Radix (HuangQi) have the strongest association. Astragali Radix (HuangQi), Tuckahoe (FuLing), Atractylodis Macrocephalae Rhizoma (BaiZhu), Princes-Feather Fruit (ShuiHongHuaZi), and Curcumae Rhizome (EZhu) have the strongest association among cirrhotic diseases. *Oldenlandia* (BaiHuaSheSheCao), Bearded Scutellaria (BanZhiLian), Curcumae Rhizome (EZhu), and Cardamom (DouKou) have the strongest association with liver cancer and are all at the core, also called the core prescriptions of the disease.

### 3.4. Collection of Common Targets

**Figure 7:**
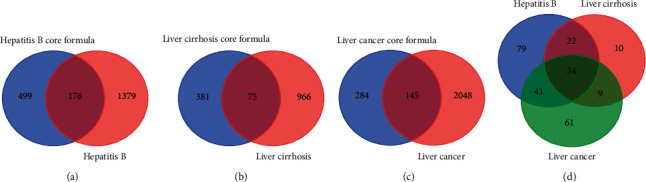
(a) Hepatitis B. (b) Cirrhosis. (c) Liver cancer. (d) Wayne diagram of the herb targets of three diseases and their core prescriptions.

The targets of the three core prescriptions were collected on TCMIP and HERB, respectively, and 675 hepatitis B targets, 456 cirrhosis targets, and 429 liver cancer targets were obtained after deduplication. Hepatitis B, cirrhosis, and liver cancer were searched on the GeneCards and OMIM databases, respectively. Score >10 was set in GeneCards screening condition, and 1,555, 1,041, and 2,193 disease targets were acquired after intersection with the obtained targets on the OMIM database, respectively. After overlapping and deduplication of the herb and disease targets with Bioinformatics and Evolutionary Genomics (https://bioinformatics.psb.ugent.be/cgi-bin/liste/Venn) (shown in Figure 7), 176, 75, and 145 common targets were obtained, respectively (see the attached file S2).

#### 3.4.1. PPI Network Diagram

**Figure 8:**
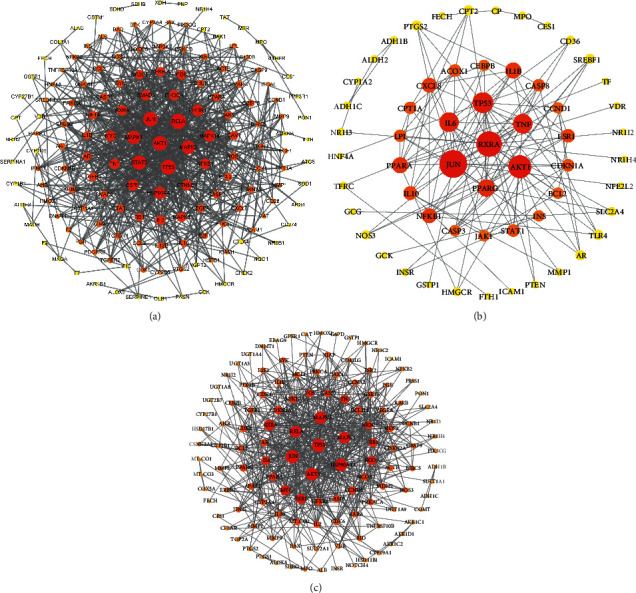
PPI network diagram. The graph was arranged in a circle under the condition of degree; those with a larger degree were placed in the center of the circle. (a) Hepatitis B. (b) Cirrhosis. (c) Liver cancer.

The minimum required interaction score in the String database was set to the highest confidence (0.9); after the free targets were filtered, there were 155 targets for hepatitis B, 58 targets for cirrhosis, and 126 targets for liver cancer. The data of the PPI network were then obtained, the file with “tsv” format was downloaded, and the PPI network diagram (shown in Figure 8) was obtained after processing with Cytoscape 3.8. The core genes of hepatitis B were AKT1, RELA, TP53, JUN, and STAT3, for cirrhosis were AKT1, JUN, and RXRA and for liver cancer were TP53, JUN, AKT1, and HSP90AA1.

### 3.5. Functional Enrichment Analysis of Core Herbs

KEGG enrichment of three interacting genes was carried out by Metascape (min overlap = 3, *P* value cut-off <0.01). The diseases-related pathways in the KEGG pathway were eliminated in order to present the pathways of the core herbs on the three chronic liver diseases directly, and a total of 116 pathways were obtained with 64 pathways for hepatitis B, 68 pathways for cirrhosis, and 96 pathways for liver cancer (see the attached file S3). Important pathway categories modulated by the prescription are listed in Table 1, and signal transduction, immune system, endocrine transduction system, cell growth, and death all played a role in the three prescriptions, in which the signal transduction and immune system pathways were the most extensive and critical ones. In the treatment of liver cancer, the nervous system-related pathways were more regulated than others. As for the cellular community system-related pathways, the core prescriptions of hepatitis B and liver cancer regulated more than what the core prescription of cirrhosis did.

**Figure 9:**
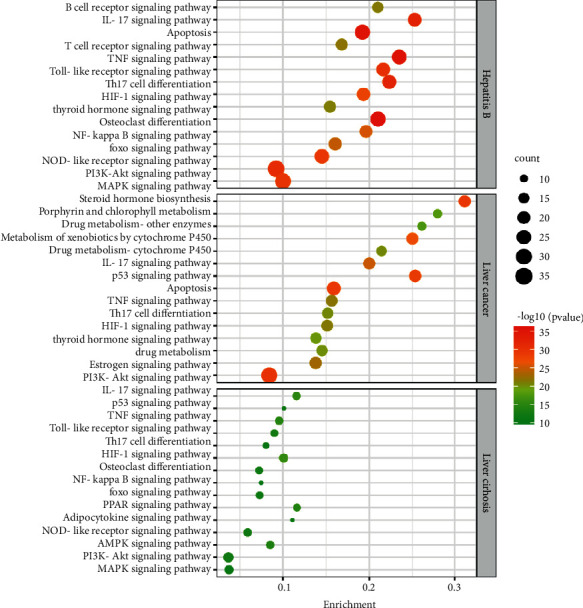
KEGG enrichment bubble plot presentation. Each bubble represents a KEGG process. Enrichment indicates the proportion of the target in the list of targets. The size of bubble is positively correlated with the number of targets in each pathway; the larger the bubble is, the richer the gene is. −log10 indicates *P* value, the greener the color is, the larger *P* value is, and a redder color indicates a smaller *P* value. The top 15 KEGG for *P* values are shown above.

The important pathways are shown in Figure 9. Among the top 15 important pathways, the important pathways cointervened by the core prescriptions of the three diseases were signal transduction-related pathways, including PI3K-Akt, TNF, and HIF-1 signaling pathways. The immune system-related pathways included Th17 cell differentiation and IL-17 signaling pathways.

**Figure 10:**
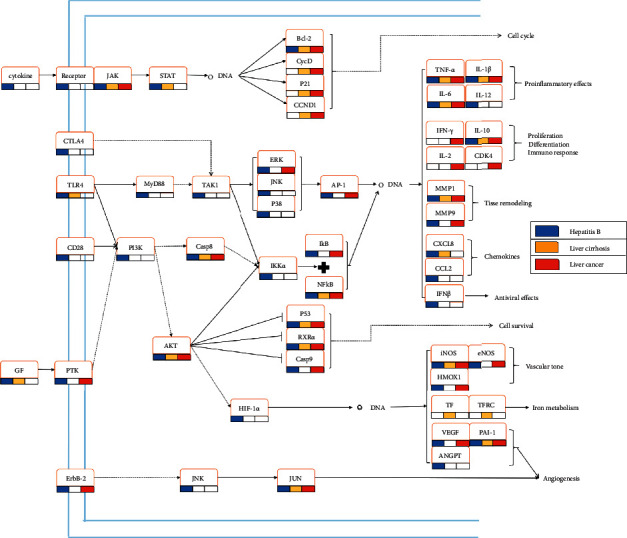
The gene regulatory effects of the core prescriptions of the three liver diseases. Light blue lines indicate cell membrane, and gene names are shown in rectangles. The markers below the genes show the aiming pattern of the three core prescriptions. Genes that lacked the upstream and downstream parts were omitted and indicated by dashed arrows.

The pathways related to signal transduction and immune system were summarized in order to explore the key pathways in detail, and some target genes that lack the upstream and downstream parts were omitted, as shown in Figure 10. TCM intervenes through a variety of ways, including the regulation of biological processes, such as cell cycle, cell survival, inflammatory response, immune response, and iron metabolism. It was found in this study that the prescription for hepatitis B played an extensive role in the related pathways, and cirrhosis prescription played a role in the middle and downstream of the pathways, mainly in inflammatory response, cell survival, cell cycle, and biological functions of iron metabolism. The liver cancer prescription mainly played a role in the middle and downstream of the pathway network, as well as in the biological functions of inflammatory response, cellular processes, angiogenesis, and vascular tension.

## 4. Discussion

Integrated analysis was used in this study to analyze the properties and flavors of herbs. In general, herbs of cold nature accounted for the greatest proportion, followed by the herbs of warm nature. The cold herbs in the prescriptions were mainly bitter in taste, and the warm herbs were mainly sweet. Salty cold herbs were slightly more prescribed in liver cirrhosis treatment. A similar proportion of herbs with other properties was prescribed for all three liver diseases. Cold herbs were related to inflammation/immunity modulation, and warm herbs affected cell growth and proliferation, reflecting that TCM herbs are widely involved in immune and cellular processes [30, 31].

In the framework of TCM theory, bitter cold herbs cleared away heat and removed pathogenic qi, and sweet warm and sweet mild herbs had a tonic effect, which suggested that removing excess substances and supplementing what lacked to restore balance were the main principle for TCM in treating the three liver diseases [32]. It can be seen from the classification of the efficacy of herbs that tonifying deficiency, clearing away heat and toxin, and activating blood circulation to remove blood stasis were the three main therapeutic approaches to liver diseases, regulating qi was emphasized in the treatment of hepatitis B, tonifying deficiency and activating blood circulation were often used for liver cirrhosis, and liver cancer was mainly treated by clearing away heat and toxin and resolving phlegm based on TCM theory. Then, the core prescriptions for the three diseases were obtained through association rules analysis. The prescriptions of the three diseases were different in that hepatitis B prescription was mainly to invigorate the spleen and soothe the liver, the liver cirrhosis prescription was mainly to invigorate qi and activate blood, and the liver cancer prescription was to remove cancer toxins while invigorating the spleen and activate blood. The three therapeutics above were the core treatments for these diseases, which were different from those in the current guidelines. The current guidelines, therapeutics, and prescriptions for the three diseases are listed in Table 2. And it is the approach of traditional Chinese medicine to treat diseases on the basis of one therapeutics while applying multiple therapeutics comprehensively [33]. There is a close relationship between hepatitis B, liver cirrhosis, and hepatocellular carcinoma. In a certain sense, they are recognized as one disease in certain stages of progression. TCM believes that patients are with more severe lesions in the blood phase than in the qi phase. The results of the analysis of patient characteristics show that, with the progression of the disease and increase of the patients' age, the disease becomes worse, which goes from the qi phase deep to the blood phase, showing that the disease is getting worse. TCM believes that [34] damp heat epidemic pathogenic factor and deficiency of vital qi are the key factors in the pathogenesis of hepatitis B, and the location of the disease is in the qi phase (shallow level). Blood stasis and phlegm are the key factors in the progression of hepatitis B into liver cirrhosis and liver cancer, and the location of the disease is in the blood phase (deep level). Compared with liver cirrhosis, phlegm and blood stasis transforming into toxin accumulation is the key to the formation of liver cancer [35], which is consistent with the core prescription treatment principles in this study.

DNA of hepatitis B virus participates directly in the onset of liver cancer through DNA integration of viral oncoproteins. At the same time, hepatocellular death and inflammatory infiltration caused by long-term infection could accelerate liver cell renewal, promote mutation accumulation, and cause liver tumors indirectly [38]. When chronic liver diseases worsened into cirrhosis, changes such as inflammation, stellate cell activation accompanied by fibrosis, angiogenesis accompanied by liver microvascular changes, and others might happen, which would increase the risk of liver cancer [7, 39, 40].

As an important factor in liver disease progression, chronic inflammation and cell growth and apoptosis are involved in the whole process of hepatitis B, liver cirrhosis, and liver cancer. The pathways related to signal transduction and the immune system were mostly intervened in the core prescriptions of the three diseases, which intervened jointly in the PI3K-Akt signaling pathway, TNF signaling pathway, HIF-1 signaling pathway, Th17 cell differentiation, and IL-17 signaling pathways, and were involved in immune regulation, inflammation, cell cycle and other important roles [41–43]. It could be seen from the PPI network interaction diagram that AKT1 was the common key target of the three prescriptions, was widely expressed in liver, which affected cell survival, proliferation, and migration when activated, and was closely related to angiogenesis [44, 45]. The PI3K-Akt signaling pathway was jointly regulated by the core prescriptions of the three diseases, which was an important inflammatory regulatory pathway, involved in cell survival and cell growth, and had a key role in tumorigenesis and development [46–48]. The hepatitis B virus core protein promoted the occurrence of liver cancer after activating the Src/PI3K/Akt pathway [49]. The largest surface antigen encoded by HBV is the large hepatitis B virus (LHBs) glycoprotein. Studies have shown that LHBs activate the Src/PI3K/Akt signaling pathway through proximal stimulation of the PKC*α*/Raf1 signaling pathway, which could promote the tumorigenesis of hepatoma cells [50]. The hepatitis B virus *X* protein-binding protein (HBXIP) may promote the proliferation and migration of liver cancer cells through the PI3K/AKT signaling pathway [51]. When there was continuous inflammation, the production of a large number of cytokines would activate the FAK-PI3K-Akt-P70 signaling cascades, result in the proliferation of HSCs, and accelerate the fibrotic response [52]. Turmeric Root Tuber (YuJin) was the most important herb in hepatitis B prescription, and its main ingredient, the essential oil of Rhizoma curcumae (EORC), significantly affected the PI3K/AKT pathway and effectively inhibited the progression of liver fibrosis [53]. Astragali Radix (HuangQi) was important in the prescriptions for hepatitis B and liver cirrhosis. Reports showed that Astragaloside IV, the main component contained in Astragali Radix (HuangQi), could improve liver damage through the PI3K/Akt pathway [54]. *Oldenlandia* (BaiHuaSheSheCao) and Bearded Scutellaria (BanZhiLian) were important herbs in the core prescription of liver cancer. They significantly induced apoptosis of cancer cells by regulating PI3K/Akt and effectively inhibited tumor growth in vitro and in vivo [55].

Apart from regulating immunity and inhibiting inflammation, the prescription for hepatitis B had a regulatory effect on the endocrine system as well, which might be an important mechanism for preventing the progression and degeneration of hepatitis B. Compared with hepatitis B prescription, liver cirrhosis prescription regulated more endocrine system pathways. Studies have found that growth hormone resistance and low insulin-like growth factor-1, thyroid dysfunction, and other problems are common in patients with liver cirrhosis, which will affect negatively its prognosis [56]. It has been found that obesity, diabetes, and fatty liver are independently associated with the increased risk of hepatocellular carcinoma [57]. The prescriptions for liver cirrhosis had a regulatory effect on the key molecules RXR and PPAR in the PPAR signaling pathway, which was involved in lipid regulation and glucose metabolism. Actively regulating hormones and maintaining stable glucose and lipid metabolism could effectively protect liver tissue and prevent liver cancer. Compared with the other two prescriptions, the liver cancer prescription regulated the most extensive pathways and regulated more cell processes and endocrine system-related pathways as well. In addition, it had a prominent regulatory effect on the nervous system. It has been found clinically that neural-related factors could significantly affect the prognosis of liver cancer, which have received increasing attention in these years [58], while the methods for liver tumors and nervous system treatment are still limited [59], for which TCM could be an important supplement.

It was found from the subnetworks (Figure 10) compiled in this study that the three prescriptions for the three liver diseases interfered extensively with the inflammatory process, and the proinflammatory cytokines IL-6, IL-1*β*, and TNF*α*, as well as the anti-inflammatory cytokine IL-6 and its upstream factor NF-*κ*B, were important targets that were regulated by the three prescriptions [60]. NF-*κ*B could be activated in almost all chronic liver diseases [61–63], which has a wide range of functions in various cell compartments, including liver cell survival, inflammation in Kupffer cells, and HSC activation, and is at the key position in chronic liver diseases and cellular wound repair response [64]. Compared with the other two prescriptions, the prescription for hepatitis B regulated more proinflammatory factors and chemokines. Another interesting finding was that hepatitis B regulated angiogenesis and VEGF pathways, while to the best of our knowledge, there has been no report on treating hepatitis B through angiogenesis intervention. Hepatocellular carcinoma is a highly vascularized tumor characterized by active neovascularization [65]. Intervention in angiogenesis before hepatitis B worsening into liver cancer might be effective in preventing the occurrence of liver tumors. The prescription for liver cirrhosis could additionally act in the biological function of iron metabolism; while there have been few studies on the application of iron metabolism in treating liver diseases, neither has this topic received enough attention. As liver is the main organ for iron storing, it is particularly vulnerable to damage from iron overload. Excessive iron produces free radicals, aggravates cell damage, leads to fibrosis, and accelerates liver cirrhosis worsening into liver cancer [66, 67]. As iron overload has often been observed in chronic liver diseases, iron metabolism would become a potential therapeutic target [68].

## 5. Innovation and Limitations

This research applied a combined method of data mining and integrated network pharmacology and explored the prescription rules and mechanism of action of TCM in the treatment of various chronic liver diseases. To the best of our knowledge, this study is the first of its kind in TCM to analyze various related diseases jointly, which has created a new paradigm in methodology for the analysis of related diseases.

With the application of this method, the core prescriptions and the herbs involved for the three liver diseases were identified, which mainly intervened in the process of chronic hepatitis B, cirrhosis, and liver cancer through the intervention of signal transduction and immune system-related pathways and regulated the immune and cell growth processes, in which the PI3K-Akt was an important signaling pathway of it. Hepatitis B prescription was helpful for angiogenesis regulation, which might be the reason why it prevented the disease progression. The prescriptions for liver cirrhosis and liver cancer interfered more with the endocrine system pathways, and the liver cancer prescription had a broader regulating effect on the nervous system. The findings may cast some light on the treatment of chronic liver diseases as well as prevention of liver cancer using TCM. The prescriptions in this study came from two professors in outpatient clinics; even though there was a lack of evaluation on the efficacy of prescriptions and the results of the analysis may be biased, the two professors are prestigious Chinese medicine experts who boast leading positions in the field of liver disease in China. Their prescriptions have been an important source of learning for doctors in the hepatology department of traditional Chinese medicine, and thus, it is still of great significance to analyze their prescriptions. In addition, the source of the cases would be expanded in future studies, and further experimental studies are required for the evaluation and validation of the action mechanisms of the core prescriptions.

## 6. Conclusion

Three core prescriptions for chronic liver disease treatment were identified in this study, and it was found that they mainly functioned through regulating signal transduction and immune system-related pathways. Compared with the other two liver diseases, the core prescription for liver cancer regulated more neural system-related pathways.

## Figures and Tables

**Table 1:** 

	Hepatitis B	Liver cirrhosis	Liver cancer
Signal transduction	20	20	22
Immune system	15	12	16
Endocrine system	7	11	13
Cell growth and death	4	5	5
Cellular community—eukaryotes	4	1	5
Nervous system	1	1	8
Other systems	13	18	27

**Table 2:** 

Disease	Source	Treatment principle	Prescription
Hepatitis B	The clinical guidelines of diagnosis and treatment of chronic hepatitis B with traditional Chinese medicine [36]	Replenish the spleen and soothe the liver, nourish the liver and kidney, promote blood circulation and free the collaterals, and clear heat and drain dampness	Yinchenhao Decoction, Xiaoyao Powder, Yiguan Decoction, and Gexia Zhuyu Decoction

Liver cirrhosis	Chinese guidelines on the management of liver cirrhosis [26]	Strengthen qi and promote blood circulation, free the network vessels and resolve the stasis, and clear heat and drain dampness	Anluo Huaxian Pill, Fuzheng Huayu Capsule, Compound Biejiaruangan Troche, and so on

Liver cancer	Standardization for diagnosis and treatment of hepatocellular carcinoma [37]	Clear the cancer toxin, replenish the spleen and promote blood circulation, soothe the liver, and nourish the blood	Xiaoyao Powder, Guipi Decoction, Huaier Granule, Cinobufagin, Jiedu Granule, and so on

## Data Availability

All the data used to support the findings of this study are available from the corresponding author upon reasonable request.

## Supplementary Materials

Supplementary Materials S1: The important association rules of the prescriptions of the three diseases were obtained via the Apriori algorithm. S2: The targets of the core herbs of hepatitis B, cirrhosis, and liver cancer. S3: KEGG enrichment analysis for the three core herbs of the three diseases.
